# Combined analysis of transcriptome and metabolite data reveals extensive differences between black and brown nearly-isogenic soybean (*Glycine max*) seed coats enabling the identification of pigment isogenes

**DOI:** 10.1186/1471-2164-12-381

**Published:** 2011-07-29

**Authors:** Nik Kovinich, Ammar Saleem, John T Arnason, Brian Miki

**Affiliations:** 1 Bioproducts and Bioprocesses, Research Branch, Agriculture and Agri-Food Canada, Ottawa, ON, Canada K1A 0C6; 2 Ottawa-Carleton Institute of Biology, Department of Biology, Carleton University, Ottawa, ON, Canada K1S 5B6; 3 Department of Biology and Center for Research in Biopharmaceuticals and Biotechnology, University of Ottawa, Ottawa, ON, Canada K1N 6N5

## Abstract

**Background:**

The *R *locus controls the color of pigmented soybean (*Glycine max*) seeds. However information about its control over seed coat biochemistry and gene expressions remains limited. The seed coats of nearly-isogenic black (*iRT*) and brown (*irT*) soybean (*Glycine max*) were known to differ by the presence or absence of anthocyanins, respectively, with genes for only a single enzyme (anthocyanidin synthase) found to be differentially expressed between isolines. We recently identified and characterized a UDP-glycose:flavonoid-3-*O*-glycosyltransferase (*UGT78K1*) from the seed coat of black (*iRT*) soybean with the aim to engineer seed coat color by suppression of an anthocyanin-specific gene. However, it remained to be investigated whether *UGT78K1 *was overexpressed with anthocyanin biosynthesis in the black (*iRT*) seed coat compared to the nearly-isogenic brown (*irT*) tissue.

In this study, we performed a combined analysis of transcriptome and metabolite data to elucidate the control of the R locus over seed coat biochemistry and to identify pigment biosynthesis genes. Two differentially expressed late-stage anthocyanin biosynthesis isogenes were further characterized, as they may serve as useful targets for the manipulation of soybean grain color while minimizing the potential for unintended effects on the plant system.

**Results:**

Metabolite composition differences were found to not be limited to anthocyanins, with specific proanthocyanidins, isoflavones, and phenylpropanoids present exclusively in the black (*iRT*) or the brown (*irT*) seed coat. A global analysis of gene expressions identified *UGT78K1 *and 19 other anthocyanin, (iso)flavonoid, and phenylpropanoid isogenes to be differentially expressed between isolines. A combined analysis of metabolite and gene expression data enabled the assignment of putative functions to biosynthesis and transport isogenes. The recombinant enzymes of two genes were validated to catalyze late-stage steps in anthocyanin biosynthesis *in vitro *and expression profiles of the corresponding genes were shown to parallel anthocyanin biosynthesis during black (*iRT*) seed coat development.

**Conclusion:**

Metabolite composition and gene expression differences between black (*iRT*) and brown (*irT*) seed coats are far more extensive than previously thought. Putative anthocyanin, proanthocyanidin, (iso)flavonoid, and phenylpropanoid isogenes were differentially-expressed between black (*iRT*) and brown (*irT*) seed coats, and *UGT78K2 *and *OMT5 *were validated to code UDP-glycose:flavonoid-3-*O*-glycosyltransferase and anthocyanin 3'-*O*-methyltransferase proteins *in vitro*, respectively. Duplicate gene copies for several enzymes were overexpressed in the black (*iRT*) seed coat suggesting more than one isogene may have to be silenced to engineer seed coat color using RNA interference.

## Background

Flavonoids represent a class of plant secondary metabolites that have evolved a variety of physiological functions including pigmentation, pathogen defense, and UV protection [1]. Additionally, metabolic engineering of flavonoids has become an important target for plant biotechnology, as flavonoids provide health benefits to foods, favorable agronomic traits to crops, and may be used in the future to color commercial transgenic materials such as grains to facilitate their identification and monitoring [2-4].

Commercial soybean (*Glycine max *(L.) Merr.) has a yellow grain. However rare spontaneous mutants exist that have black (*iRT*) or brown (*irT*) seed coat (testa) color phenotypes. Black (*iRT*) and brown (*irT*) soybean seed coats contain proanthocyanidins (PAs, a.k.a. condensed tannins) but differ in the presence/absence of anthocyanins [5]. A goal for biosafety is to engineer a novel red seed coat color as a marker for transgenic soybean grains to facilitate their identification [4], and could potentially be achieved by the suppression of anthocyanin-specific genes that are overexpressed in the black soybean seed coat. However the genes have not yet been identified.

Six genetic loci (*I*, *R*, *T*, *Wp*, *W1*, and *O*) [6] identified by classical genetics control flavonoid-based seed coat color in soybean. The *I *locus controls the presence or absence and spatial distribution of flavonoid pigments and has four alleles (*I*, *I^i^*, *I^k^*, *i*); *I *gives completely non-pigmented seed coat, *I^i ^*restricts pigment to the hilum and *I^k ^*to a saddle-shaped region, whereas the *i *allele results in a fully pigmented seed coat [6]. The recessive *i *allele results from spontaneous deletion of *CHS4 *or *CHS1 *promoter sequences and results in the abolishment of a posttranscriptional RNA silencing mechanism that results in the increased accumulation of chalcone synthase (CHS) transcripts in the seed coat [7,8].

The *T *locus is a pleoiotropic locus that controls the type and abundance of flavonoid pigments in the seed coat in addition to other traits such as seed coat cracking and trichome pigmentation [5,9,10]. Genetic polymorphisms that affect the expression or function of the flavonoid 3'-hydroxylase gene (*F3'H1*) have been shown to co-segregate with recessive *t *alleles [11,12].

The *W1 *locus controls flower color and affects seed color only in an *iRt *background; where *W1 *and *w1 *alleles give imperfect black and buff seed coat colors, respectively [6]. The *W1 *allele for purple flower color was shown to code flavonoid 3',5'-hydroxylase (F3'5'H), as a 65-bp insertion in the gene (*F3'5'H*) co-segregated with white flower color (*w1*) [13].

The Wp locus was suggested to code the flavonone 3-hydroxylase gene (*F3H1*) by microarray analysis as high levels of *F3H1 *transcripts co-segregated with purple flower color (*Wp*), and low levels with pink (*wp*) flowers [14]. In the seed coat, recessive *wp *resulted in a change from black (*iRTWp*) to a lighter grayish (*iRTwp*) color [14].

The *O *locus affects the color of brown (*irTO*) seed coats, with the recessive *o *allele giving a red-brown (*irTo*) phenotype [6]. The *O *locus has been suggested to code the proanthocyanidin (PA, a.k.a. condensed tannin) biosynthesis gene anthocyanidin reductase (ANR), as the gene was located between markers that flank the *O *locus on the soybean physical map [15]. However molecular genetics analyses have not yet demonstrated the identity of the *O *locus gene.

Finally, the *R *locus controls the presence (*R*) or absence (*r*) of anthocyanins in black (*iRT*) or brown (*irT*) seed coats, respectively [16]. Despite the identification of several pigment genes from soybean, only transcripts for anthocyanidin synthase (ANS) genes (*ANS23-1*, *ANS27-1 *and *ANS100*) have been shown to be overexpressed in the black (*iRT*) seed coat compared to the brown (*irT*) nearly-isogenic line by northern blot [17]. The upregulation of several ANS genes suggests the *R *locus to code a regulatory factor, and raises the possibility that other isogenes for anthocyanin biosynthesis may be upregulated.

Recently, a cDNA coding the UDP-glycose:flavonoid-3-*O*-glycosyltransferase (UF3GT) gene (*UGT78K1*) was isolated from the black (*iRT*) seed coat and shown to function in anthocyanin biosynthesis *in vitro *and by complementation of a gene mutation in Arabidopsis [18]. However *UGT78K1 *expressions have not been investigated in relation to seed coat color.

The soybean genome sequence Glyma1 was predicted to code 46,430 protein-coding genes with nearly 75% of the genes present in multiple copies [19]. This may suggest a relatively high frequency of functional redundancy and increased difficulty in identifying soybean genes by traditional approaches. However, using transcriptome analysis tools such as the Soybean GeneChip equipped with 37,500 probe sets in combination with broad-coverage metabolite analysis methodologies such as LC-MS/MS, gene functions could potentially be efficiently predicted. The combined analysis of transcriptome and metabolite data has been shown to be a powerful approach for the functional identification of unknown genes [20-22]. Metabolite differences caused by the overexpression of a transcription factor, the exposure to a nutritional stress, and by species differences have been correlated with differences in transcriptome profiles to successfully predict the functions of unknown genes in flavonoid, glucosinolate, and alkaloid biosyntheses [21-23].

In the present study we have employed targeted and non-targeted metabolite analysis methodologies and have demonstrated that black (*iRT*) and brown (*irT*) nearly-isogenic soybean seed coats do not just differ in the presence/absence of anthocyanins, and have extensive differences in procyanidin, (iso)flavonoid and phenylpropanoid compositions. The underlying differences in gene expressions were then identified by microarray analysis, and the putative functions of 20 unknown genes were assigned by comparison to metabolite data. From the set of differentially regulated genes, two putative late-stage anthocyanin genes were selected and the functions of their coded enzymes were validated *in vitro*. In addition, a set of gene candidates potentially coded by the R locus have been provided.

## Results

### qRT-PCR indicates differential expressions of anthocyanin and proanthocyanidin genes in nearly-isogenic black (*iRT*) and brown (*irT*) soybean seed coats

Among the flavonoid genes identified from soybean seed coats, only *ANS *transcripts were found to be overexpressed in the seed coat of black (*iRT*) soybean compared to the brown (*irT*) isoline, however no RFLP difference was observed when probing *ANS *genes [17]. This suggests that *ANS *overexpression is not the only molecular difference between black (*iRT*) and brown (*irT*) seed coats. To examine whether other anthocyanin genes are upregulated, transcript profiles of the recently identified *UGT78K1 *[18] and the *DFR1 *gene [GenBank:AF167556] were measured from seed coat cDNA by quantitative RT-PCR (qRT-PCR) using *ANS2*/*ANS3 *transcripts [GenBank:AY382829/GenBank: AY382830] as a positive control. Phosphenol pyruvate carboxylase (*PEPC*) [GenBank: D10717] was used as an endogenous reference for normalization of the qRT-PCR measurements of target genes across seed coat samples, as PEPC is expressed at similar levels in a wide range of soybean tissues and has been used previously as a reference for gene expressions during soybean seed coat development [8,24].

**Figure 1:**
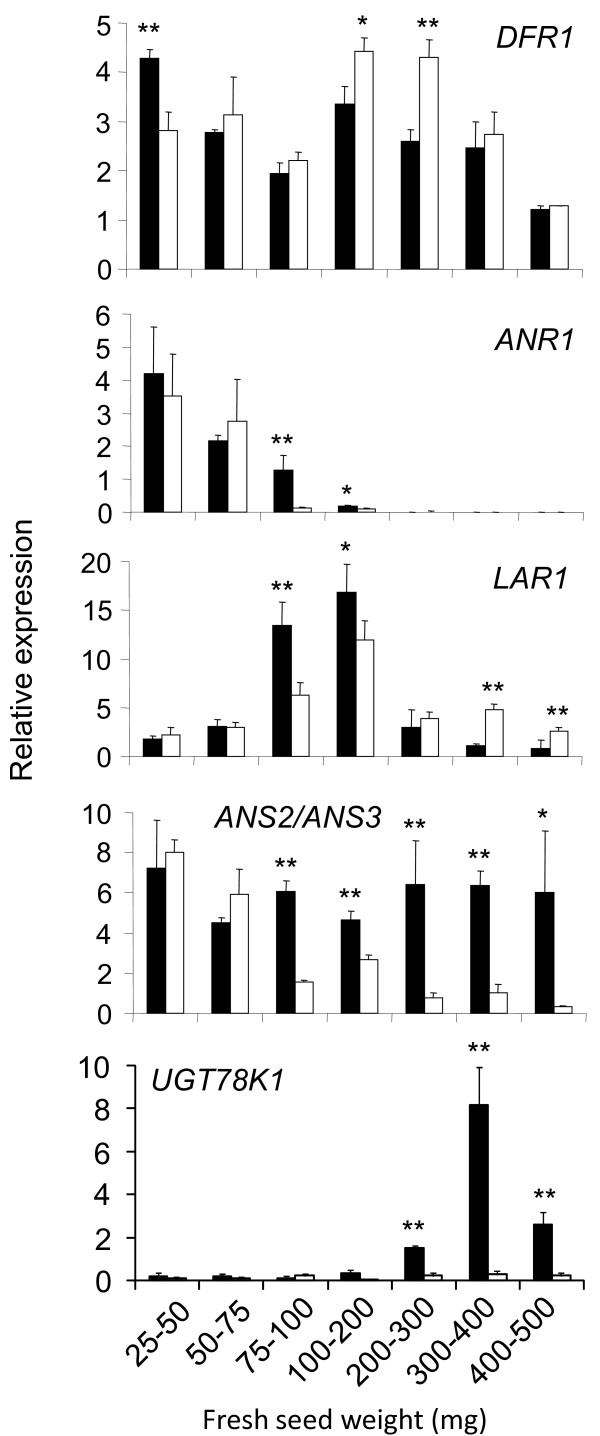
**Transcript profiles from black (*iRT*) and brown (*irT*) seed coats during development measured by qRT-PCR**. Black (*iRT*) soybean (black bars) and brown (*irT*) soybean (white bars). Student's *t *test significant at P < 0.01 (**), student's t test significant at P < 0.05 (*).

**Figure 2:**
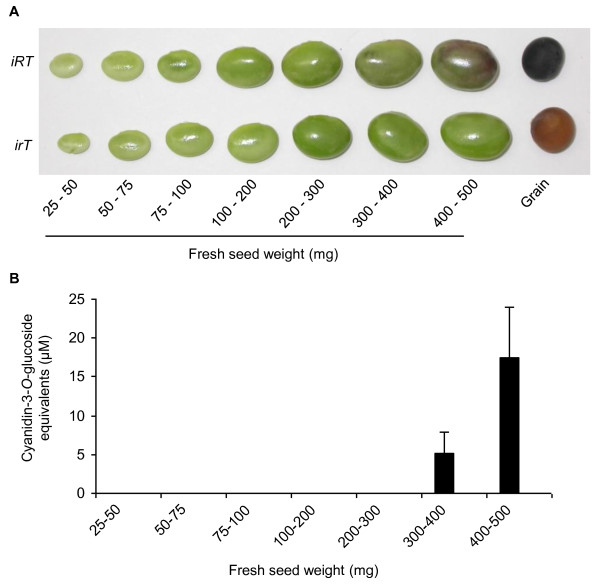
**Anthocyanin accumulations in black (*iRT*) and brown (*irT*) soybean seed coats during seed development**. The fresh weight stages of seed development are indicated. (A) Seed color phenotypes during development. (B) Total amounts of anthocyanins in the seed coat of black (*iRT*) soybean during seed development. No anthocyanin absorbance was measured from brown (*irT*) seed coat extracts.

qRT-PCR demonstrated *UGT78K1 *to be significantly overexpressed in the black (*iRT*) seed coat at the transition stage (300 mg - 400 mg FSW) and early maturation stage (400 - 500 mg FSW) of seed development compared to the brown (*irT*) isoline, and to be almost undetectable at earlier stages (Figure 1). Stereomicroscopy and photospectroscopic measurements confirmed these developmental stages to coincide with the stages of anthocyanin biosynthesis in the black (*irT*) seed coat (Figure 2). In contrast to *UGT78K1*, *DFR1 *was not differentially expressed at the stages of anthocyanin biosynthesis (Figure 1). *ANS2*/*ANS3 *mRNAs were confirmed to be overexpressed in the black (*iRT*) seed coat at most stages (Figure 1) as shown previously [17].

The seed coat palisade cells of black (*iRT*) soybean are unusual compared to those of other model plants used for the study of anthocyanin biosynthesis (e.g. Arabidopsis, Maize, and Petunia) because black (*iRT*) soybean palisade cells synthesize both anthocyanins and proanthocyanidins (PAs). ANR and LAR enzymes have been identified to catalyze the conversion of cyanidin to epicatechin and leucocyanidin to catechin, respectively, which are the putative initial biochemical steps of PA biosynthesis [25,26]. To investigate how a seed tissue may coordinate the biosynthesis of these parallel pigment pathways, the expressions of putative PA genes *ANR1 *[GenBank:JF433915] and *LAR1 *[GenBank:JF433916] were examined in relation to anthocyanin gene expressions. qRT-PCR demonstrated *ANR1 *expressions to decrease throughout seed coat development in both genotypes and to be almost undetectable by the stages of *UGT78K1 *overexpressions and anthocyanin biosynthesis (Figure 1). By contrast, *LAR1 *expressions were downregulated at the stages of anthocyanin biosynthesis in the black (*iRT*) seed coat (Figure 1).

In conclusion, these results show that the recently identified *UGT78K1 *is upregulated in the black (*iRT*) seed coat, in addition to *ANS2*/*ANS3*, and that there is a simultaneous downregulation of the PA gene *LAR1 *at the stages of anthocyanin biosynthesis. These results demonstrate that expression differences between black (*iRT*) and brown (*irT*) seed coats are not limited to anthocyanin genes, and may suggest extensive differences in gene expressions and phenolic compositions.

### Combined analysis of targeted and non-targeted methodologies indicate overaccumulation of anthocyanins, altered procyanidin, and reduced flavonol, benzoic acid, and isoflavone compositions in the seed coat of black (*iRT*) soybean

The structures of anthocyanins from only a few (Korean) black soybean varieties have been described [27,28] and information regarding other phenolic compositions may be limited to proanthocyanidins (PAs), where acid-catalyzed hydrolysis of black (*iRT*) and brown (*irT*) seed coats showed the PAs to be 3',4'-hydroxylated [5]. A more extensive analysis of phenolic composition differences between black (*iRT*) and brown (*irT*) seed coats could greatly improve our understanding of seed pigmentation, and may suggest gene activities that function in pigment biosynthesis. For these reasons, black (*iRT*) and brown (*irT*) Clark seed coat metabolites were extracted at the transition stage (300 mg - 400 mg FSW) of seed development with multiple solvents and analyzed by targeted high performance liquid chromatography-diode array detection (HPLC-DAD) and non-targeted HPLC-tandem mass spectrometry (HPLC-MS/MS). To ensure seed coats were at the same stage of development, the days post anthesis, pod length, pod color, embryo morphology, and transcript levels of the developmental marker gene Gm-r1083-1191, a putative cutin biosynthesis gene [29], and the metabolism gene *DFR1 *were ensured to be equivalent between black (*iRT*) and brown (*irT*) tissues (Additional file 1: Table S1).

**Figure 3:**
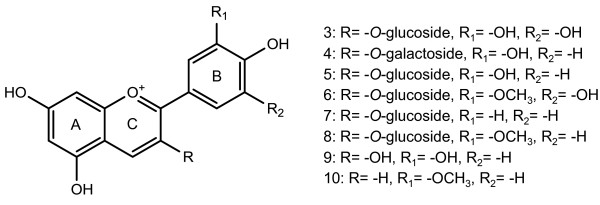
**Anthocyanins from the seed coat of black (*iRT*) soybean variety Clark**. Numbers correspond to chromatographic peaks identified by HPLC-DAD (see Table 1).

For a targeted analysis of anthocyanins, black (*iRT*) and brown (*irT*) Clark seed coats were extracted with acidified aqueous methanol and metabolites were identified using HPLC-DAD by measuring the absorbance at 520 nm in comparison to authentic standards. Ten cyanic pigments were identified, the most abundant were the anthocyanins cyanidin-3-*O*-glucoside (peak 5, 43.6% total peak area), cyanidin-3-*O*-galactoside (peak 4, 17.4% peak area) and petunidin-3-*O*-glucoside (peak 6, 14.6% total peak area) with lesser amounts of delphinidin-, pelargonidin-, and peonidin-3-*O*-glucosides (peaks 3, 7, 8; 8.9%, 1.2%, and 1.1% peak areas, respectively), the aglycone cyanidin (peak 9, 3.9% peak area), the 3-deoxyanthocyanidin diosmetinidin (peak 10, 2.0% peak area), and the two unidentified anthocyanin-like compounds (peaks 1 and 2, 1.6% and 0.8% peak areas, respectively) (Table 1). To our knowledge this is the first report of a 3-deoxyanthocyanidin from the soybean seed coat. The structures of all identified anthocyanins are represented in Figure 3. By contrast, cyanic pigments were not detected from the brown (*irT*) seed coat at any stage of seed development (not shown).

**Table 1:** HPLC-DAD analysis of anthocyanins from the seed coat of black (iRT) soybean variety Clark

Peak	Rt (min)	Area (%) (520 nm)	Identity*
1	1.7	1.6	unidentified compound 1
2	2.6	0.8	unidentified compound 2
3	5.5	8.9	delphinidin-3-*O*-glucoside
4	6.3	17.4	cyanidin-3-*O*-galactoside
5	6.7	43.6	cyanidin-3-*O*-glucoside
6	7.1	14.6	petunidin-3-*O*-glucoside
7	7.4	1.2	pelargonidin-3-*O*-glucoside
8	7.7	1.1	peonidin-3-*O*-glucoside
9	9.5	3.9	Cyaniding
10	9.7	2.0	diosmetinidin

*For chemical structures see Fig. 3.

To compare the structural features of PAs, black (*iRT*) and brown (*irT*) seed coats were extracted with 70% acetone and subjected to phloroglucinol cleavage analysis [30,31]. PA free monomers, polymer subunit compositions, and mean degree of polymerization (mDP) did not differ significantly between nearly-isogenic black (*iRT*) and brown (*irT*) seed coats. The soluble PA mDP was 4, and the subunit and monomer compositions consisted mainly of epicatechin with minor amounts of unidentified compounds (Additional file 2: Figure S1A - S1C). Reaction with DMACA reagent demonstrated similar amounts of soluble PAs from both black (*iRT*) and brown (*irT*) seed coats (P = 0.08) (Additional file 2: Figure S1F). Further extraction of seed coat residues at 50°C in the presence of acid and phloroglucinol yielded only low levels of epicatechin-phloroglucinol adduct, epicatechin terminal units, and several unidentified compounds (Additional file 2: Figure S1B), and acid catalyzed cleavage of seed coat residues at 95°C identified relatively high, but equivalent amounts of 3',4'-hydroxylated solvent insoluble PAs (Additional file 2: Figure S1E).

**Figure 4:**
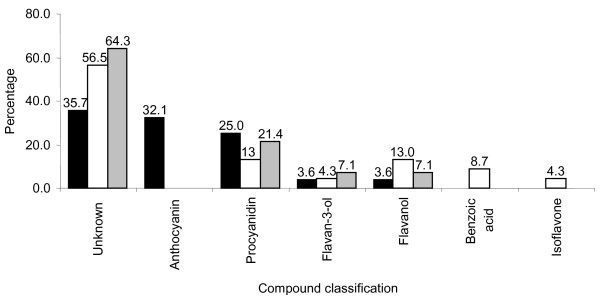
**Distribution of compound classes from black (*iRT*) and brown (*irT*) soybean seed coats identified by LC-MS/MS**. *iRT*, black bars; *irT*, white bars; common to both genotypes, grey bars.

For a non-targeted analysis of soluble phenolic metabolites, nearly isogenic black (*iRT*) and brown (*irT*) Clark seed coats were extracted with acidified aqueous methanol and analyzed by HPLC-MS/MS over a mass range of 100 amu to 1000 amu. Identities were assigned to peaks by comparison of retention times and mass fragmentation patterns to authentic standards. Peaks with no corresponding authentic standards were identified by mass fragmentation patterns in comparison to the literature. A total of 177 peaks were detected, of which 78 peaks were assigned to 37 compounds (Table 2). Twenty-eight compounds were detected from the black (*iRT*) seed coat (Table 2); consisting mainly of unidentified compounds (35.7%) and anthocyanins (32.1%), followed by procyanidins (25.0%). By contrast, 23 compounds were detected from the brown (*irT*) seed coat (Table 2); consisting mainly of unidentified compounds (56.5%), followed by flavonols (13.0%) and procyanidins (13.0%). The majority of compounds (62.2% of all compounds identified by HPLC-MS/MS) were exclusive to the black (*iRT*) or brown (*irT*) seed coat, suggesting extensively different biochemical specializations of these tissues. The distribution of compound classes from black (*iRT*) and brown (*irT*) Clark seed coats is illustrated in Figure 4.

**Table 2:** HPLC-MS/MS analysis of seed coat extracts of black (iRT) and brown (irT) soybean

Compound	Identity	[M^+^] (m/z)	Major fragment(s)	Rt (min)	Genotype^a^
1	Unknown 1	413	183, 115	1.4	B
2	Unknown 2	381	184, 125	1.6	*irT*
3	Unknown 3	183	-	1.7	*iRT*
4	Unknown 4	162	-	1.9	B
5	Unknown 5	409	303	1.9	B
6	Unknown 6	437	273	2.3	B
7	Unknown 7	416	-	2.3	B
8	Unknown 8	580	182, 265, 437	2.5	B
9	Unknown 9	507	485, 323, 132	2.9	B
10	Orcinol *O*-hexoside	305	287	4.0	*irT*
11	Unknown 10	331	248	4.2	*irT*
12	Procyanidin trimer 1	867	579, 309, 299	4.7	*iRT*
13	Procyanidin B2*	579	291	5.5	B
14	Unknown 11	607	409, 455	6.0	B
15	Procyanidin trimer 2	867	577, 407	6.4	B
16	Delphinidin-3-*O*-glucoside*	465	303	6.6	*iRT*
17	Procyanidin trimer 3	867	579	6.6	*iRT*
18	Epicatechin*	291	-	6.9	B
19	Cyanidin-3-*O*-galactoside*	449	287	7.1	*iRT*
20	Daidzein *O*-hexoside malonylated	503	417	7.4	*irT*
21	Unknown 12	481	319	7.4	*irT*
22	Cyanidin-3-*O*-glucoside*	449	287	7.5	*iRT*
23	Petunidin-3-*O*-glucoside*	479	319, 303	8.0	*iRT*
24	Unknown 13	341	278, 111	8.1	*irT*
25	Pelargonidin-3-*O*-glucoside*	433	-	8.3	*iRT*
26	Peonidin-3-*O*-glucoside*	463	301, 286	8.7	*iRT*
27	Procyanidin dimer 2	579	409, 291	9.2	B
28	Unknown flavonol 1	331	-	9.0	*irT*
29	Procyanidin trimer 4	867	579, 699	9.2	*iRT*
30	A-type procyanidin dimer	593	-	9.7	*iRT*
31	Unknown anthocyanin 1	317	-	9.6	*iRT*
32	Unknown flavonol 2	615	-	9.6	*irT*
33	Cyanidin*	287	-	10.0	*iRT*
34	Unknown 14	487	-	10.8	B
35	Diosmetinidin*	285	-	10.3	*iRT*
36	Unknown benzoic acid 1	633	331, 315	11.0	*irT*
37	Quercetin-3-O-glucoside*	465	303	11.0	B^b^

*Based on co-chromatographic and MS fragamentation comparison to an authentic standard.

^a^Black soybean (*iRT*), brown soybean (*irT*), both (B).

^b^Trace amounts in *iRT*.

The 14 compounds exclusive to the black (*iRT*) seed coat were anthocyanins (8 compounds), procyanidins (4 compounds), and unknown compounds (2 compounds) (Table 2). HPLC-MS/MS (Table 2) confirmed the identity of anthocyanins found by HPLC-DAD (Table 1). Procyanidin tetramers and larger oligomers were not detected as they exceeded the mass range (1000 amu) analyzed by the HPLC-MS/MS experiments, however 3 PA trimers (Procyanidin trimer 1, 2, and 4) and the A-type procyanidin dimer were exclusive to the black (*iRT*) seed coat (Table 2).

The 9 compounds exclusive to the brown (*irT*) seed coat consisted of 4 unknown compounds (Unknown compound 2, 10, 12, and 13), 2 flavonols (Unknown flavonol 1 and 2), 2 benzoic acids (Orcinol *O*-hexoside and Unknown benzoic acid 1), and the isoflavone daidzein *O*-hexoside malonylated (Table 2).

Taken together, our metabolite analysis experiments show that nearly-isogenic black (*iRT*) and brown (*irT*) Clark seed coats have compositional differences not limited to the presence or absence of anthocyanins; with specific flavonol, isoflavone, and benzoic acids exclusive to the brown (*irT*) seed coat and anthocyanins and unique procyanidin oligomers exclusive to the black (*iRT*) seed coat. These results may suggest extensive differences in the underlying gene expressions.

### Microarray Analysis Indicates Concomitant Upregulation of Specific Phenylpropanoid, Flavonoid, and Anthocyanin Isogenes and Downregulation of Other Flavonoid, Benzoic Acid, and Isoflavone Isogenes in the Black (*iRT*) Seed Coat Transcriptome

Nearly 75% of genes in the soybean genome are present in multiple copies [19], and previously only transcripts for *ANS *isogenes were found to be overexpressed in the black (*iRT*) seed coat compared to the nearly-isogenic brown (*irT*) tissue [17]. For a global analysis of isogene expressions, black (*iRT*) and brown (*irT*) Clark seed coats were dissected at the transition stage of seed development (300 - 400 mg FSW) for microarray analysis using the Affymetrix Soybean GeneChip. Seed coats were ensured to be at the same stage of development as described above for the metabolite analyses (Additional file 1: Table S1).

Of the 37,500 probe sets present on the Soybean GeneChip, 80 were found to be upregulated more than 2-fold in black (*iRT*) soybean (Additional file 3: Table S2). Classification using the Gene-Bins ontology tool (http://bioinfoserver.rsbs.anu.edu.au/utils/GeneBins/index.php) demonstrated the majority of upregulated genes to be "unclassified without homology" (28.7%) or "unclassified with homology" (24.8%), followed by "biosynthesis of secondary metabolites" (7.9%) (Additional file 4: Figure S2). This latter class consisted exclusively of putative (and some characterized) phenylpropanoid, flavonoid, and anthocyanin isogenes. Interestingly, putative isogenes of the phenylpropanoid pathway (4CL-like and 4CL2), the flavonoid pathway (CHS4/5, F3H-like, DFR2, ANS2/ANS3), and the late-stage anthocyanin pathway (UGT78K1, UGT78K2, OMT-like, OMT5, GST21 and GST26) were all upregulated more than 2-fold in the black (*iRT*) seed coat, more than 234-fold in the case of the GST26 probe set (Table 3).

**Table 3:** Putative phenylpropanoid/flavonoid gene sets upregulated more than 2-fold in the seed coats of black (iRT) soybean compared to the brown (irT) isoline^a,b^.

					SAM	
					
Gene Family	Probe Sets	Name	Target Description	Black/brown fold change	P-value	d-score
Phenyl-propanoid/Flavonoid pathway	GmaAffx.80720.1.S1_at	DFR2*	Dihydroflavonol-4-reductase	62.45	0.000002	24.70
	GmaAffx.42116.1.S1_at	CHS4*/CHS5*	Chalcone synthase (LOC732575)	10.99	0.000020	8.45
	GmaAffx.42116.1.S1_x_at			8.06	0.000026	7.124398
	Gma.1163.1.S1_at	ANS2*/ANS3*	Anthocyanidin synthase	6.77	0.000018	8.77
	Gma.17141.1.S1_at	F3H-like	Moderately similar to A. thaliana putative flavonone 3-β-hydroxylase clone U20744 (74%)	6.43	0.004849	2.03
	Gma.7423.2.S1_a_at	4CL-like	4-coumarate-CoA ligase-like protein	2.80	0.005815	1.96
	Gma.5621.1.S1_at	G4DT*	Pterocarpan 4-dimethylallyltransferase	2.36	0.009232	1.80
	Gma.8472.1.S1_at	4CL2*	4-Coumarate-CoA ligase 2	2.27	0.000108	4.60
Glycosyl-transferase	GmaAffx.71999.1.S1_at	UGT78K2	Glycosyltransferase	3.09	0.000124	4.50
	Gma.1002.2.S1_at	UGT78K1*	UDP-glucose:flavonoid 3-*O*-glucosyltransferase	2.81	0.000229	4.03
O-methyl-transferase	Gma.9647.1.S1_at	OMT-like	O-methyltransferase, putative	2.58	0.000098	4.74
	GmaAffx.57777.1.S1_at	OMT5	Caffeoyl-CoA *O*-methyltransferase 5	2.48	0.000157	4.33
Glutathione S-transferase	GmaAffx.71212.1.A1_at	GST26	Glutathione S-transferase	234.11	0.000003	21.22
	Gma.5139.1.S1_at	GST21	Glutathione S-transferase	2.05	0.000656	3.09

^a^Expression values were obtained using RMA (Irizarry et al., 2003).

^b^See Table S2 for the complete list of upregulated genes.

*Previously characterized flavonoid biosynthesis genes.

A BLASTP search of the soybean genome sequence Glyma1 (http://www.phytozome.net/soybean) using the conserved plant secondary product glycosyltransferase signature sequence (PSPG box) [32] identified 214 annotated Family 1 glycosyltransferases (UGTs)(Additional file 5: Table S3). Of the 214 UGTs, only 2 were found to be upregulated more than 2-fold from the black (*iRT*) seed coat; the recently identified UF3GT gene *UGT78K1 *[18] was upregulated more than 2.8-fold and a second uncharacterized glycosyltransferase, which we named *UGT78K2 *in accordance with UGT nomenclature [33], was upregulated more than 3-fold (Table 3).

A search of the soybean genome sequence with the conserved OMT-domain sequence (PLN02589; NCBI) and the ontology '*O*-methyltransferase', identified 84 annotated OMTs (Additional file 6: Table S4). Of the 84 OMT genes, only probe sets for OMT-like and OMT5 were upregulated in the black (*iRT*) seed coat, more than 2.4-fold and 2.5-fold, respectively (Table 3).

A total of 52 probe sets were downregulated more than 2-fold in the black (*iRT*) seed coat (i.e. black/brown < 0.5 fold); among these, 2 probe sets by more than 10-fold, 12 probe sets by more than 3-fold, with the remainder between 2- and 3-fold (Table 4; Additional file 7: Table S5). Classification of down-regulated genes using the Gene-Bins ontology tool demonstrated the majority to be "unclassified without homology" (45.5%) or "unclassified with homology" (12.1%), followed by "biosynthesis of secondary metabolites" (9.1%) (Additional file 8: Figure S3). The latter class consisted primarily of characterized and putative (iso)flavonoid isogenes. Isogenes for common flavonoid biosynthesis (CHS1, CHS9, F3'H), PA biosynthesis (LAR1), and isoflavone biosynthesis (IFR1-like) were all down-regulated more than 2-fold in the black (*iRT*) seed coat, more than 35-fold in the case of the CHS1 (Table 4). The flavonoid genes CHS9 and F3'H were down-regulated more than 21-fold and 2.5-fold, respectively, the putative isoflavone gene IFR1-like by more than 3.6-fold, and the putative PA gene LAR1 by more than 2.2-fold (Table 4).

**Table 4:** Putative phenylpropanoid/flavonoid gene probe sets downregulated more than 2- fold in the black (iRT) compared to the brown (irT) soybean seed coat^a,b^

Gene Family					SAM	
					
	Probe Sets	Name	Target Description	Black/brown fold change	P-value	d-score
Phenyl-propanoid/	Gma.17605.1.S1_at	CHS1*	Chalcone synthase	0.028	0.000007	-16.45
Flavonoid	GmaAffx.92491.1.S1_s_at	CHS9*	Chalcone synthase	0.047	0.000005	-19.36
pathway	GmaAffx.89828.1.S1_s_at	IFR1-like	Highly similar to isoflavone reductase homolog 1 (IFR1) (93%)	0.272	0.000132	-4.46
	Gma.8455.1.S1_at	sf3'h1*	Flavonoid 3'-hydroxylase	0.400	0.000646	-3.10
	GmaAffx.93221.1.S1_s_at	OOMT-like	Moderately similar to Rosa rugosa orcinol O-methyltransferase (OOMT-A) (72%)	0.410	0.003428	-2.17
	GmaAffx.34868.1.A1_at	LAR1	Highly similar to Phaseolus coccineus leucoanthocyanidin reductase (LAR) (88%)	0.443	0.000016	-9.02
	Gma.4340.2.S1_a_at		Moderately similar to P. trichocarpa flavonoid O-methyltransferase-related (73.9%)	0.462	0.004180	-2.08
	Gma.10949.1.S1_s_at		Moderately similar to A. thaliana 3-chloroallyl aldehyde dehydrogenase/aldehyde dehydrogenase (NAD)/coniferyl-aldehyde dehydrogenase (ALDH2C4) (75.5%)	0.495	0.000273	-3.92
Transport	GmaAffx.39265.1.S1_at	MATE1	Weakly similar to A. thaliana antiporter/drug transporter MATE family protein (69.8%)	0.414	0.000265	-3.95

^a^Expression values were obtained using RMA (Irizarry et al., 2003).

^b^See Table S5 for the complete list of up-regulated genes.

*Previously characterized flavonoid biosynthesis genes.

Of the differentially regulated probe sets, 12 represented transcription factor genes, 8 were upregulated and 4 downregulated (Additional file 3: Table S2 and Additional file 7 Table S5, respectively), however none of these genes clustered with known flavonoid transcription factor orthologs by phylogenetic analysis (not shown).

**Figure 5:**
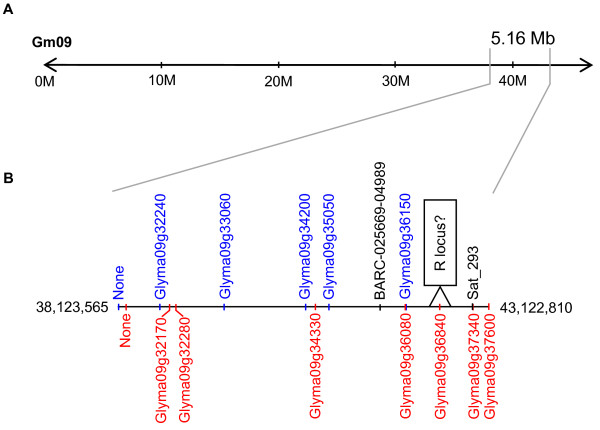
**Genomic organization of differentially regulated genes from chromosome Gm09 of the Glyma1 sequence**. (A) All differentially expressed genes were located in a 5.16 Mb region of the distal end of chromosome Gm09. (B) Enlargement of the 5.16 Mb region showing the locations of the upregulated genes (blue) and downegulated genes (red) relative to sequence-based markers BARC-025669-04989 and Sat_293 (black) that are situated upstream and downstream, respectively, nearest the R locus in the Glyma1 sequence. Chromosome positions of differentially regulated genes are listed in Table S6. Chromosome positions of BARC-025669-04989 (Gm09:41,620,379..41,620,751) and Sat_293 (Gm09:42,848,788..42,849,068).

A previous study mapped the R locus between markers A668_1 and K387_1 on MLG K (Chromosome 9) [34]. Nineteen differentially-regulated probe sets were associated with genes located on chromosome 9 of the soybean genome sequence Glyma1 (Additional file 9: Table S6). Interestingly, all genes were located in a 5.16 Mb region of the distal arm of chromosome 9, and only the downregulated AP2/ERF (Glyma09g36840) and serine carboxypeptidase-like protein (Glyma09g36080) and the upregulated polyamine oxidase (Glyma09g36150) were located between markers (BARC-025669-04989 and Sat_293) that flank the putative position of the R locus (http://soybeanphysicalmap.org) (Figure 5).

To validate the microarray results, 28 differentially-regulated genes were selected and their relative expression profiles were measured by semi-quantitative RT-PCR (semi-qRT-PCR) using black (*iRT*) and brown (*irT*) seed coat cDNA as templates. Phosphenol pyruvate carboxylase (PEPC) was used as the endogenous control to ensure equal loading and the flavonoid genes *CHS7*/*CHS8 *and the putative PA gene *ANR1 *were used as negative controls as they were not found to be differentially expressed in the microarray analysis. Semi-qRT-PCR confirmed the differential-expressions of all 28 genes and demonstrated similar expressions for *CHS7*/*CHS8 *and *ANR1 *(Additional file 10: Figure S4). Furthermore, as the upregulated probe set GmaAffx.42116.1.S1 did not distinguish between *CHS4 *and *CHS5 *expressions, semi-qRT-PCR was performed with gene specific primers to show the *CHS4 *gene, and not the *CHS5 *gene to be upregulated in the black (*iRT*) seed coat (Additional file 10: Figure S4).

In summary, these results show that numerous specific anthocyanin, flavonoid and phenylpropanoid isogenes are upregulated and other flavonoid, benzoic acid, and isoflavone isogenes are downregulated in the black (*iRT*) seed coat transcriptome relative to that of the nearly-isogenic brown (*irT*) tissue.

### Putative Assignment of Function to Genes Differentially-Regulated between Black (*iRT*) and Brown (*irT*) Seed Coats

**Figure 6:**
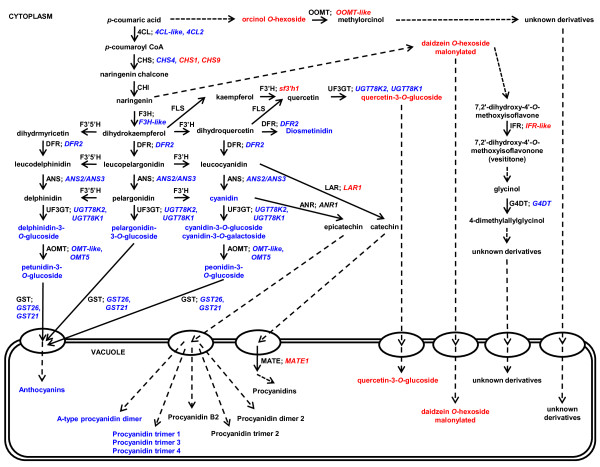
Summary of flavonoid/phenylpropanoid gene and metabolite accumulations from black and brown soybean seed coats*. Transcripts upregulated and metabolites exclusive to the black (*iRT*) seed coat are shown in blue. Transcripts downregulated and metabolites absent in the black (*iRT*) seed coat but present in the nearly-isogenic brown (*irT*) seed coat are shown in red. 4CL, 4-coumarate-CoA ligase; OOMT, orcinol O-methyltransferase; CHS, chalcone synthase; CHI, chalcone isomerase; F3H, flavanone 3-hydroxylase; F3'H, flavonoid 3'-hydroxylase; F3'5'H, flavonoid 3',5'-hydroxylase; DFR, dihydroflavonol reductase; ANS, anthocyanidin synthase; UF3GT, UDP-glycose:flavonoid 3-*O*-glycosyltransferase; AOMT, anthocyanin *O*-methyltransferase; GST, glutathione *S*-transferase; FLS, flavonol synthase; LAR, leucoanthocyanidin reductase; ANR, anthocyanidin reductase; MATE, multidrug and toxic extrusion protein; IFR, isoflavone reductase; G4DT; pterocarpan 4-dimethylallyltransferase. *For the complete lists of differentially regulated transcripts see Table S2 and Table S5, and for a complete list of differentially accumulated metabolites see Table 2.

Gene functions can be successfully predicted based on a combined analysis of transcriptome and metabolite data [21]. We compared differences in gene expressions determined by microarray to differences in metabolite compositions to assign putative functions to genes from the black (*iRT*) and brown (*irT*) seed coats (Figure 6). In addition to 7 previously characterized soybean flavonoid genes, several uncharacterized genes belonging to large multi-gene families with possible roles in anthocyanin biosynthesis and transport were upregulated; including one glycosyltransferase (UGT78K2), two *O*-methyltransferases (OMT5 and OMT-like) and two glutathione *S*-transferases (GST21 and GST26) (Table 3). Considering the accumulation of metabolites with specific molecular structures in the black (*iRT*) seed coat, putative functions of the differentially-expressed genes were assigned to specific structural modifications and transport functions (Figure 6).

Anthocyanins in the seed coat of black (*iRT*) soybean are glycosylated at the 3-position of the C-ring (Figure 3). The glycosyltransferase UGT78K2 is assigned to code a UDP-glycose:flavonoid 3-*O*-glycosyltransferase (UF3GT) protein (Figure 6) and may have functional redundancies in anthocyanin biosynthesis with the previously characterized UGT78K1 [18], which was also upregulated in the black (*iRT*) seed coat (Table 3). Two anthocyanins (petunidin-3-*O*-glucoside and peonidin-3-*O*-glucoside) and one deoxyanthocyanidin (diosmetinidin) from the black (*iRT*) seed coat are methylated at the 3-position of the B-ring (the 3'-position) (Figure 3). The two upregulated *O*-methyltransferases were assigned to code anthocyanin 3'-*O*-methyltransferase (AOMT) proteins (Figure 6) and may have redundant function in anthocyanin biosynthesis this tissue.

### *UGT78K2 *and *OMT5 *Code UDP-Glycose:Flavonoid 3-*O*-Glycosyltransferase and Anthocyanin 3'-*O*-Methyltransferase Proteins, Respectively

**Figure 7:**
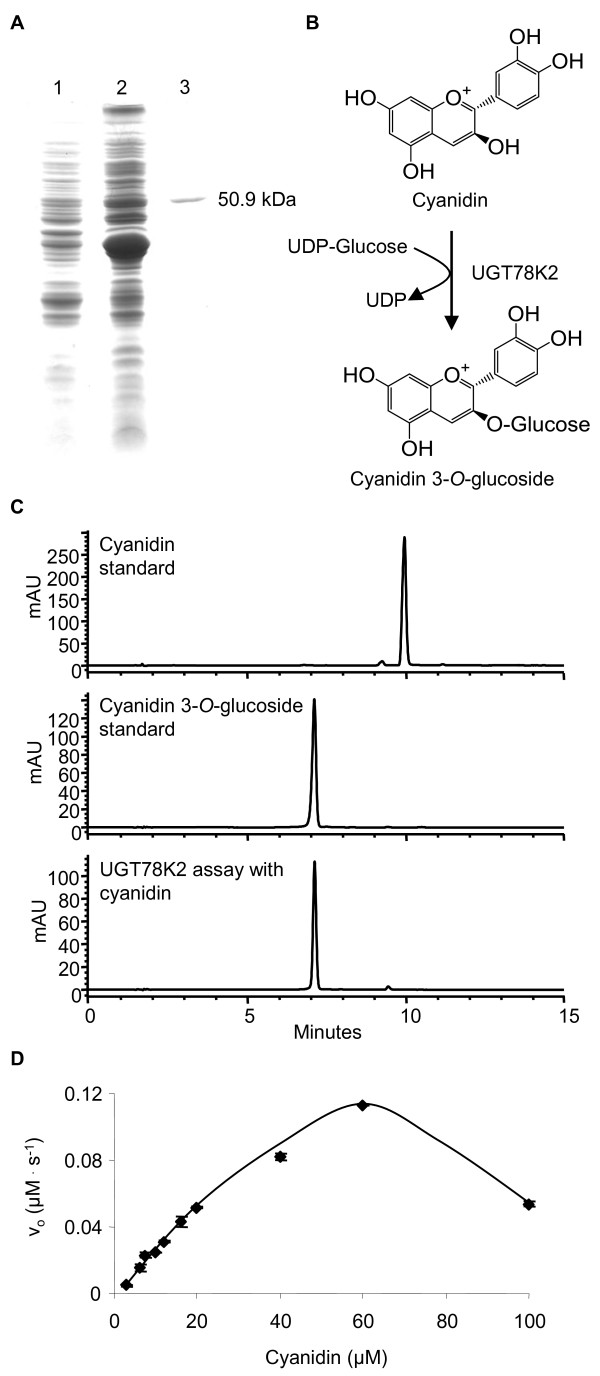
**Purification of his-tagged UGT78K2 and determination of the reaction product and kinetics for cyanidin**. (A) SDS-PAGE analysis of UGT78K2: total soluble protein from E. coli expressing UGT78K2 prior to induction with 100 lM IPTG (lane 1), 24 h post-induction (lane 2); IMAC-purified UGT78K2 (lane 3). (B) The UGT78K2 enzyme reaction as revealed by HPLC-DAD chromatograms at 520 nm (C). HPLC retention times: cyanidin (Rt: 10.1 min); cyanidin 3-*O*-glucoside (Rt: 7.1 min). (D) UGT78K2 kinetics for the cyanidin acceptor substrate using 5 mM UDPG as a sugar donor. Points represent the mean of three assays ± the standard deviation.

Reducing the expressions of late-stage anthocyanin genes by RNA interference (RNAi) in the seed coat of black soybean may be an effective strategy to engineer seed coat color while minimizing unintended effects on other flavonoid subpathways. However, functional redundancies may require that multiple genes be silenced in order to reduce enzyme activity enough to reduce pigment levels. Two glycosyltransferases (*UGT78K1 *and *UGT78K2*) were upregulated in the black (*iRT*) seed coat relative to the nearly-isogenic brown (*irT*) tissue (Table 3) and the upregulation paralleled the accumulation of anthocyanins with 3-*O*-glycosylated structures (Figure 2), suggesting the function of these genes as UDP-glycose:flavonoid 3-*O*-glycosyltransferases (UF3GTs). We have characterized UGT78K1 previously and shown it to possess UF3GT activity towards anthocyanidins *in vitro *and *in vivo *[18]. To determine the catalytic function of *UGT78K2 *[GenBank:HM591298], the ORF was cloned into an N-terminal hexahistidine fusion tag vector and expressed in *E*. *coli *for analysis of the coded recombinant protein *in vitro*. The recombinant protein (rUGT78K2) was purified by ion-metal-affinity chromatography (IMAC) had an apparent mass of 50.9 kDa that matched well with the calculated mass of the native UGT78K2 protein (49.04 kDa) in addition to the 2.04 kDa N-terminal hexahistidine fusion tag encoded by the pET-14b vector (Figure 7A). In assays containing UDP-glucose as the sugar donor and cyanidin as the acceptor, the recombinant enzyme transferred glucose to the 3-position of cyanidin to form cyanidin-3-*O*-glucoside (Figure 7B, C), whereas the boiled enzyme and bacteria expressing the corresponding empty vector could not catalyze this reaction (not shown). Similarly, in assays containing UDP-galactose as the sugar donor and cyanidin as the acceptor, the recombinant enzyme transferred galactose to the 3-position of cyanidin to form cyanidin-3-*O*-galactoside (not shown). Cyanidin-3-*O*-glucoside and cyanidin-3-*O*-galactoside from recombinant enzyme assays were identified by HPLC-DAD in comparison to the authentic standards, and confirmed by HPLC-MS/MS with the characteristic parent ions 449.0 [cyanidin-3-*O*-glucoside or cyanidin-3-*O*-galactoside]^+1 ^and the MS/MS fragment 287.3 [cyanidin]^+1 ^corresponding to the loss of glucose (-162 amu).

As we have performed the kinetics analysis for recombinant UGT78K1, we were able to employ the same experimental conditions to test the kinetics of UGT78K2. Interestingly, UGT78K2 converted cyanidin-3-*O*-glucoside with greater velocity than UGT78K1 and higher concentrations of cyanidin were required to inhibit its activity (Figure 7D) despite the enzymes having 93% amino acid similarity (Additional file 11: Figure S5).

**Figure 8:**
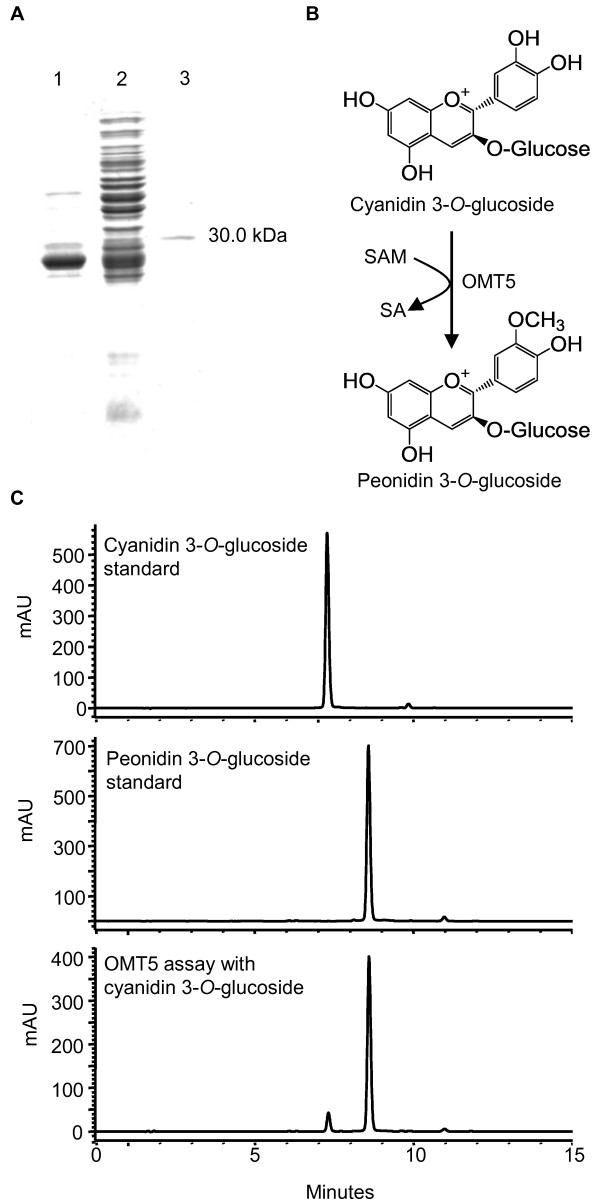
**Purification of his-tagged OMT5 and determination of the reaction product for cyanidin 3-*O*-glucoside**. (A) SDS-PAGE analysis of OMT5: total soluble protein from E. coli expressing OMT5 prior to induction with 100 lM IPTG (lane 1), 24 h post-induction (lane 2); IMAC-purified OMT5 (lane 3). (B) The OMT5 enzyme reaction as revealed by HPLC-DAD chromatograms at 520 nm (C). HPLC retention times: cyanidin 3-*O*-glucoside (Rt: 7.3 min); peonidin 3-*O*-glucoside (Rt: 8.6 min).

Methylation of the B-ring of anthocyanins has a reddening effect on flower color (reviewed by [35]) and thus engineering the expression of 3'-*O*-methyltransferases could change the redness of seed color. However, despite the identification of several flavonoid OMTs from soybean, none have been shown to accept anthocyanins as substrates. Our microarray analysis identified two *O*-methyltransferases (OMT5 and OMT-like) to be upregulated in the black (*iRT*) seed coat (Table 3) and the upregulation paralleled the accumulation of 3'-*O*-methylated anthocyanins in this tissue (Figure 2), suggesting the function of these genes as anthocyanin 3'-*O*-methyltransferases (AOMTs). Following the approach described above, the ORF of *OMT5 *[GenBank:HQ856048] was cloned for expression and functional characterization of the corresponding recombinant protein *in vitro*. The purified recombinant protein had an apparent mass of 30.0 kDa that matched well with the calculated mass (27.78 kDa) in addition to the 2.04 kDa N-terminal hexahistidine fusion tag encoded by the pET-14b vector (Figure 8A). In assays containing S-adenosyl-L-methionine (SAM) as the methyl donor and cyanidin-3-*O*-glucoside as the acceptor, the recombinant enzyme transferred a methyl group to the 3'-position of the acceptor to form peonidin-3-*O*-glucoside (Figure 8B, C), whereas the boiled enzyme and bacteria expressing the corresponding empty vector could not catalyze this reaction (not shown). Peonidin-3-*O*-glucoside from recombinant enzyme assays was identified by HPLC-DAD in comparison to the authentic standard, and confirmed by HPLC-MS/MS with the characteristic parent ion 463.1 [peonidin-3-O-glucoside]^+1 ^and the MS/MS fragment 301.0 [peonidin]^+1 ^corresponding to the loss of glucose (-162 amu).

**Figure 9:**
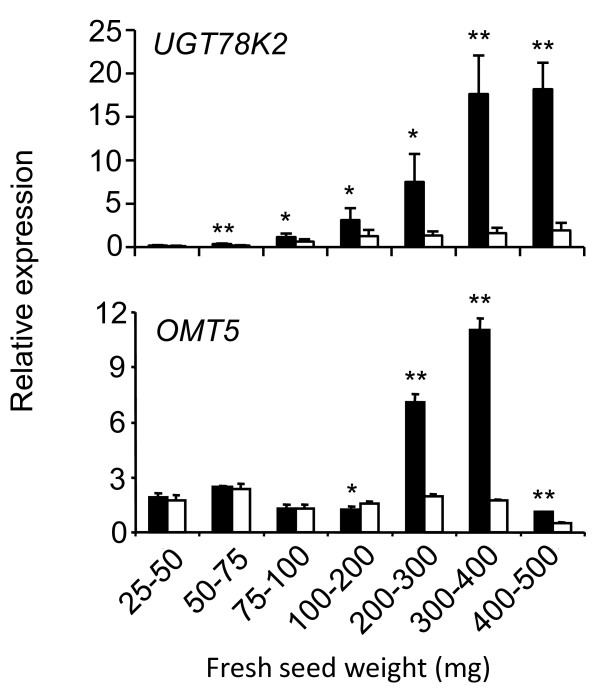
**Seed coat transcript profiles of *UGT78K2 *and *OMT5 *during seed development measured by qRT-PCR**. Black (*iRT*) seed coat (black bars) and brown (*irT*) seed coat (white bars). Student's *t *test significant at P < 0.01 (**), student's t test significant at P < 0.05 (*).

To further investigate the function these genes, *UGT78K2 *and *OMT5 *expression profiles were measured from seed coat cDNA throughout black (*iRT*) and brown (*irT*) soybean seed development by quantitative RT-PCR (qRT-PCR) (Figure 9) and expressions were compared to anthocyanin accumulation profiles throughout seed development (Figure 2). Similar to *UGT78K1*, *UGT78K2 *and *OMT5 *expressions peaked at the transition and early maturation stages of seed development (300 mg - 400 mg FSW, 400 mg - 500 mg FSW) in the black (*iRT*) seed coat (Figure 9), the stages at which anthocyanins accumulate (Figure 2), and were low or almost undetectable at early stages of seed development in the black (*iRT*) seed coat and at all stages in the brown (*irT*) seed coat.

Together the qRT-PCR and recombinant enzyme assays suggest that *UGT78K2 *and *OMT5 *code UF3GT and AOMT proteins, respectively, that function in anthocyanin pigment biosynthesis in the seed of black (*iRT*) soybean.

## Discussion

The seed coat compositions of black (*iRT*) and brown (*irT*) soybean (*Glycine max*) were known to differ only by the presence/absence of anthocyanins, however our metabolite analysis demonstrated these nearly-isogenic tissues to have extensive differences in anthocyanin, proanthocyanidin (PA), (iso)flavonoid, and phenylpropanoid content (Table 2). Of the 37 compounds detected, 24 compounds (62.2% of all compounds identified by HPLC-MS/MS) were exclusive to either the black (*iRT*) or the brown (*irT*) seed coat. Analysis of the distributions of compound classes between seed coats demonstrated the black (*iRT*) to a have a greater proportion of anthocyanins and PAs, and the brown (*irT*) to have a greater proportion of flavonol, benzoic acid, isoflavone, and unknown metabolite compositions (Figure 4). Metabolites exclusive to the black (*iRT*) seed coat included PA trimers (Procyanidin trimer 1, 2, and 4) and an A-type procyanidin dimer in addition to various anthocyanins (Table 2).

The anthocyanins identified from variety Clark were mainly similar to those reported previously from various Korean varieties [27,28] however Clark was the first shown to contain a 3-deoxyanthocyanidin (diosmetinidin) in the seed coat (Table 2). 3-deoxyanthocyanidins are relatively rare intensely coloured pigments found in *Sorghum bicolour *in response to fungal attack [36], and from the cotyledons of yellow soybean in response to UV-C treatment [37]. The biosynthesis of 3-deoxyanthocyanidins from flavonones has been shown to be a minor activity of a DFR isogene in Sorghum [38]. It would be interesting to determine whether the DFR gene that was highly overexpressed in the black Clark seed coat (*DFR2*) had similar catalytic activity, and whether the Korean varieties contain genetic alterations related to *DFR2*.

Analysis of the black (*iRT*) seed coat transcriptome compared to that of the nearly-isogenic brown (*irT*) tissue identified 80 probe sets to be upregulated (Table 3). This is almost double the number of probe sets upregulated by overexpression of the MYB transcription factor PAP1 in Arabidopsis (38 probe sets) [21], similar to the number upregulated by overexpression of the anthocyanin factor LAP1 in *M. truncatula *(61 probe sets), and less than half the number upregulated by overexpression of LAP1 in *M. sativa *(270 probe sets) [39]. The higher numbers of genes upregulated in anthocyanin-overaccumulating tissues of black soybean, *M. truncatula *and *M. sativa *likely reflect the fact that legumes have undergone genome duplication and that genes with multiple copies are frequently retained [19]. Our data supported this hypothesis as duplicate copies of several genes were upregulated in the black (*iRT*) seed coat (e.g. *4CL2 *and *4CL-like*, *UGT78K2 *and *UGT78K1*, *OMT5 *and *OMT*-*like*, and *GST21 *and *GST26*).

Functional redundancies can complicate gene identification by traditional approaches. Previous to the present study, genes for only 5 flavonoid enzymes (CHS, F3H, F3'H, F3'5'H, and UF3GT) were identified to be involved in pigment biosynthesis in the soybean seed coat [7,11-14,18]. In the present study, 20 isogenes for 14 proteins were identified to have putative roles in flavonoid biosynthesis in black (*iRT*) or brown (*irT*) seed coats using a combined analysis of microarray and metabolite data (Figure 6).

Transcriptome analysis has previously demonstrated a critical role of *CHS7 *and *CHS8 *genes for isoflavonoid biosynthesis in developing embryos of soybean [40]. However, our microarray and semi-quantitative RT-PCR data demonstrated that *CHS7 *and *CHS8 *were not differentially regulated in black (*iRT*) and brown (*irT*) seed coats (Table 3). Interestingly, it was *CHS4 *that was upregulated and *CHS1 *and *CHS9 *that were downregulatedin the black (*iRT*) seed coat (Table 3 and Table 4 respectively). As *CHS4 *is upregulated with anthocyanin genes, it likely has a role in anthocyanin biosynthesis in this tissue. Consistent with this, spontaneous mutations of the dominant *i^i ^*allele to the recessive *i *allele have been shown to affect the promoter region of the *CHS4 g*ene [7]. By contrast, the downregulation of *CHS1 *and *CHS9 *may suggest that they have a role in the parallel downregulated biochemical pathways, such as flavonol and isoflavonoid biosynthesis, as these metabolites, and transcript levels of genes for their biosynthesis, were reduced or not present in the black (*iRT*) seed coat (Figure 6). These results emphasize the complexity of isogene expressions that exist to achieve a single enzyme function in soybean.

Microarray analysis identified differentially regulated probe sets for only 3 gene functions (LAR, GST, and MATE) that have been previously shown to be involved in PA biosynthesis and transport. Recombinant LAR enzymes from several legume species have been shown to convert leucocyanidin to (+)-catechin *in vitro *[25,26,41,42]. However, the *in vivo *role of LAR in PA biosynthesis remains questionable as endogenous expression has been shown not to correlate with PA levels in *M. truncatula *and heterologous expression has failed to increase PA accumulations [41]. Similarly, our metabolite data showed that soybean PAs lack catechin subunits (Additional file 2: Figure S1) and that *LAR1 *expressions (Figure 1) do not parallel PA accumulations during seed development (not shown). Interestingly, 4 PAs were found to be exclusive to the black (*iRT*) seed coat (Table 2) however the total subunit compositions, the mean degree of polymerization (mDP), and the total amounts of soluble and solvent-insoluble PAs were not different compared to the brown (*irT*) seed coat (Additional file 2: Figure S1). These results suggest the PA oligomers of black (*iRT*) and brown (*irT*) seed coats may differ only in their subunit linkages. However, the potential influence of LAR or other PA genes on PA subunit linkages (if any) remains to be determined.

From the set of differentially regulated genes identified by microarray, two putative late-stage anthocyanin genes were selected and the functions of their coded enzymes were determined *in vitro*. *UGT78K2 *was found to code a UF3GT enzyme for anthocyanin biosynthesis (Figure 7). A UF3GT cDNA (*UGT78K1*) has recently been isolated from the black seed coat and functionally characterized [18]. *UGT78K2 *was 93% similar to *UGT78K1 *but possessed increased catalytic activity towards cyanidin (Figure 7D). The identification gene redundancy has important implications for seed coat color engineering by RNA interference, as both copies of the gene may have to be silenced to achieve a significant reduction in UF3GT activity. Similarly, two OMT genes (*OMT5 *and *OMT-like*) were found to be overexpressed in the black seed coat (Table 3). *OMT5 *was cloned and found to code an AOMT enzyme *in vitro *(Figure 8). UF3GT and OMT genes may be ideal candidates for seed coat color engineering as suppression of UF3GT genes (*UGT78K1 *and *UGT78K2*) may result in a reduction in anthocyanin levels similar to that caused by a T-DNA insertion in the Arabidopsis UF3GT [21] and upregulation of an AOMT may result in a more red seed coat, as methylation of the B-ring of anthocyanins has a reddening effect on flower color (reviewed by [35]). However, it remains to be determined whether altering the expressions of these genes could be use to modify seed coat color without causing unintended effects on alternative biochemical pathways.

Prior to the present study, currently known soybean genes did not map between markers A668_1 and K387_1 on chromosome 9 of the soybean physical genomic map, where the R locus is located, leaving no obvious candidates for the R locus gene [15]. In our study, 14 genes identified to be differentially regulated in the black (*iRT*) soybean seed coat transcriptome were found to be located on chromosome 9 of the soybean genome sequence Glyma1 (Figure 5). Among the gene families represented, only WD40 and MYB have known roles in flavonoid regulation. However the genes demonstrated did not cluster with known flavonoid regulators by phylogenetic analysis (not shown). A Mob1/phocein gene was commonly upregulated in the black (*iRT*) soybean seed coat and Arabidopsis seedlings overexpressing PAP1 [21]. Other regulatory gene families represented on chromosome 9 were AP2/ERF, serine/threonine protein kinase, calcium/calmodulin-dependent protein kinase, and ethylene-responsive element-binding protein. Interestingly, all upregulated and downregulated genes on chromosome 9 were located in 5.16 Mb region that includes the nearest sequence markers to the R locus. Only the downregulated AP2/ERF transcription factor, the serine carboxypeptidase gene, and the gene moderately similar to the *A. thaliana *polyamine oxidase ATPAO1 were located between these sequence markers (Figure 5). As a result our study provides a short list of gene candidates that may serve as the focus of future efforts to identify the R locus gene.

## Conclusion

Metabolite composition and gene expression differences between black (*iRT*) and brown (*irT*) seed coats are far more extensive than previously thought. Putative anthocyanin, proanthocyanidin, (iso)flavonoid, and phenylpropanoid isogenes were differentially-expressed between black (*iRT*) and brown (*irT*) seed coats, and *UGT78K2 *and *OMT5 *were validated to code UDP-glycose:flavonoid-3-*O*-glycosyltransferase and anthocyanin 3'-*O*-methyltransferase proteins *in vitro*, respectively. Duplicate gene copies for several enzymes were overexpressed in the black (*iRT*) seed coat suggesting more than one isogene may have to be silenced to engineer seed coat color using RNA interference.

## Methods

### Chemicals

Cyanidin was purchased from Indofine (Somerville, NJ, USA) UDP-glucose and SAM from Sigma-Aldrich (Oakville, ON, CA), the 3-*O*-glucosides of delphinidin and petunidin from Polyphenols (Hanaveien, NO), and all other phytochemicals were purchased from Extrasynthese (Lyon, FR). All solvents for LC-MS/MS and HPLC-DAD analyses were of HPLC grade purchased from Fisher Scientific (Ottawa, ON, Canada).

### Plant Materials and Growth Conditions

Black (*iRT*) soybean (*G. max *(L.) Merr.) variety Clark (PI547438) and the nearly isogenic brown (*irT*) soybean (PI 547475) were obtained from the U.S. Department of Agriculture Soybean Germplasm Collection (Agricultural Research Service, University of Illinois at Champaign-Urbana). Seeds were surface-sterilized in 2% triton/70% EtOH-H2O (7:3, v/v) for 5 min on a mixer wheel, rinsed three times with EtOH, dried, and germinated in autoclaved vermiculite in a Conviron E15 cabinet with a photoperiod of 16 h light (590 μE m^2 ^s) at 25°C, and 8 h dark at 20°C. Plants were fertilized twice weekly with N:P:K 35-5-10 (1% w/v). After 12 days, seedlings were transplanted to autoclaved soil and 34 days later the photoperiod was changed to 12 h light/12 h dark to encourage reproductive development. For RNA isolation, seed coats were dissected from plants, immediately frozen in liquid N2, lyophilized, and stored at -80°C. Seed coats for comparative analyses were harvested on the same days, 4 h after the initiation of the light cycle to minimize for potential differences in transcript and metabolite accumulations that may be influenced by circadian rhythm.

### Seed Coat Metabolite Analyses

Lyophilized seed coats (~40 mg) from each developmental stage were pulverized in HCO2H-MeOH-H2O (400 μL, 15:80:5, v/v) using a FastPrep FP120 Homogenizer (Savant) and incubated at 4°C for 2 h. The slurry was centrifuged at 20,000 g for 10 min, and 5 μl of supernatant from each sample was measured by photospectroscopy using a NANODROP 2000 (Thermo Scientific) according to the formula A530-0.25A657 to compensate for chlorophyll absorption at 530 nm (Mancinelli, 1990). The quantity of anthocyanins was determined by comparison to a standard curve and expressed as cyanidin 3-*O*-glucoside equivalents. For HPLC-DAD and LC-QTRAP analyses samples were extracted for an additional 24 h and 200 μl of supernatant was vortexed with HCO_2_H-MeOH (1 mL, 15:85, v/v) for 20 sec, centrifuged at 20,000 g for 10 min, and the supernatant was evaporated under a stream of nitrogen gas (to 50 μL), filtered through PVDF (0.45 μm; Millipore), and 20 μl and 5 μl aliquots were analyzed by HPLC-DAD and HPLC-MS/MS respectively, as described below.

For analysis of proanthocyanidins (PAs), lyophilized seed coats were ground to a powder, extracted with 70% acetone (1 mg mL^-1^) and analyzed as described previously [30,31] with (-)-epicatechin, (+)-catechin, and (-)-epicatechin gallate used as standards for the identification of free monomers, and procyanidin B2 used as a standard for the analysis of soluble and insoluble PA oligomer compositions. Quantitation of soluble PAs by reaction with DMACA reagent and insoluble PAs by acid-catalyzed cleavage was performed as described [43] using procyanidin B2 and cyanidin as standards for the respective assays.

### Microarray Analysis, Semi-qRT-PCR, and qRT-PCR

Total RNA was isolated from pigmented soybean seed coats as described previously (Wang and Vodkin, 1994). The quantity and purity of RNA samples were determined by spectrophotometry using a NANODROP 2000 (Thermo Scientific). RNA integrity was confirmed by microfluidics using a Bioanalyser 2100 equipped with an RNA 6000 Nano Chip (Agilent Technologies Inc., Montreal, QC, Canada).

Microarray was performed on RNA isolated from three plants of each genotype. Briefly, 100 ng of total RNA was *in vitro *transcribed for 16 h using the GeneChip 3'IVT Express Kit (Affymetrix, http://www.affymetrix.com). Labelled RNA was hybridized to GeneChip Soybean Genome Arrays as described by the manufacturer (Affymetrix). Statistical analyses of data for the identification of differentially regulated genes were performed using FlexArray software (M. Blazejczyk and associates; Genome Quebec, Montreal) that uses R and Bio-Conductor [44]. The raw data was adjusted for background signal, and normalized across all GeneChips using the Robust Multi-array Average (RMA) method [45]. To identify differentially expressed genes, the SAM algorithm, which minimizes variation across arrays and incorporates an estimate of false discovery rate (FDR) [46], was used. Only genes with a fold change > 2 or < 0.5 and a *P *value < 0.01 were considered to be differentially expressed. All materials and procedures comply with the MIAME standards for array data [47]. The full dataset has been deposited in the Gene Expression Omnibus [GEO:GSE26208].

For semi-quantitative RT-PCR (semi-qRT-PCR), RNA samples (3 μg) were treated with DNase I (Amplification grade, Invitrogen) at 37°C for 15 min to remove contaminating DNA. Reactions (20 μl) were terminated by heating (65°C for 10 min) in the presence of 1 ul of 25 mM EDTA. First-strand cDNA was synthesized using SuperScript III Reverse Transcriptase (Invitrogen) according to the manufacturer's instructions. Parallel reactions were performed in the absence of Superscript III to test for genomic DNA contamination. Gene expressions from each cDNA sample were normalized to the endogenous reference PEPC16. Semi-quantitative RT-PCR (semi-qRT-PCR) reactions (20 μl) consisted of 2 μl of first-strand cDNA (or untreated RNA), 400 nM primers, 200 μM dNTPs, 1.5 mM MgCl_2_, 1X Taq Buffer, 2.5 units Taq polymerase (Fermentas). PCR cycling was 94°C for 2 min, followed by 23, 25, and 27 cycles for CHS7/CHS8 or 27, 30, 33 cycles for all other genes, 94°C for 15 sec, 58°C for 1 min, and 72°C 1 min.

For quantitative RT-PCR (qRT-PCR), cDNA template was prepared as described above. Reactions (25 μl) consisted of 2 μl of first-strand cDNA (or untreated RNA), 250 nM of forward and reverse primers, and 12.5 μl of the iQ SYBR Green Supermix (BioRad). qRT-PCR of each target gene for each seed coat sample was performed in triplicate on cDNA samples or untreated RNA samples using an PTC-200 Peltier thermal cycler equipped with a Chromo4 continuous fluorescence detector (MJ research). PCR cycling was 95°C for 10 min, followed by 40 cycles of 95°C for 30 sec, 58°C for 1 min, and 72°C 1 min. qRT-PCR data and PCR efficiencies were analyzed using the Opticon Monitor 3 software (BioRad). To verify the specificity of the RT-PCR reactions, melting curves were performed subsequent to each reaction in addition to fractionation of RT-PCR products on agarose gels. Semi-qRT-PCR and qRT-PCR experiments were performed in triplicate. Primers used in this study can be found in Additional file 12: Table S7.

### Cloning of UGT78K2 and OMT5 cDNAs from the Seed Coat of Black Soybean

To clone the full-length coding sequences (CDSs) of UGT78K2 and OMT5, PCR primers were designed based on the transcript sequence predicted for Glyma08g07130 (http://www.phytozome.net/soybean) and based on the mRNA sequence of BT098523, respectively. Full-length CDSs for UGT78K2 and OMT5 were amplified from black (*iRT*) seed coat cDNA by end-to-end PCR using primers UHF/UAR and HO5F/HO5R, respectively (Additional file 12: Table S7) and a high-fidelity DNA polymerase (Invitrogen). The resulting UGT78K2 and OMT5 amplicons (1466 bp and 836 bp, respectively) were cloned into NdeI and BamHI sites, and NdeI and XhoI sites of the pET-14b vector (Novagen), respectively, and sequenced to confirm their identities.

### Expression of Recombinant Proteins in *E*. *coli*

The full-length CDSs of UGT78K2 and OMT5 were independently fused in-frame to the N-terminal hexahistidine tag encoded by the pET-14b vector (above). Plasmids were transformed into the expression host *E*. *coli *BL21(DE3) pLysS (Novagen) and a single colony for each plasmid was selected for production of recombinant proteins. Soluble recombinant proteins were isolated following growth and induction of *E*. *coli *BL21(DE3) pLysS at 16°C. Recombinant proteins were purified by ion metal-affinity chromatography (IMAC) using a kit (Novagen). To confirm the purity of the recombinant enzyme, the eluted fractions were visualized on 12.5% acrylamide gel stained with 0.25% Coomassie Blue. The amount of purified recombinant enzyme was determined using the BioRad reagent.

### Recombinant Enzyme Assays

The UGT78K2 assay (total volume 100 μl) was performed as described previously [18] and consisted of recombinant enzyme (2 μg), cyanidin (20 μM), and UDPG (5 mM) in assay solution. The OMT 5 assay (total volume 200 μl) consisted of recombinant enzyme (3 μg), SAM (100 μM), MgCl_2 _(150 μM), Tris pH 7.5 (50 mM), and 2-mercaptoethanol (14 mM). UGT78K2 and OMT 5 assays were incubated for 5 min at 30°C and 1 h at 35°C, respectively. Reactions were stopped by vortexing in MeOH (500 μl) for 20 s. Reaction products were identified by comparison to commercial standards dissolved in MeOH. Reactions were prepared for HPLC by centrifugation (21,000 g for 4 min at 4°C) followed by concentration of the supernatant to final volume of 50 μl under a stream of N2 gas. All reactions were filtered through PTFE (0.45 μm; Millipore, MA USA) and 20 μl aliquots were analyzed by HPLC-DAD. Assays were performed in triplicate and experiments repeated thrice.

### HPLC-DAD and HPLC-MS/MS Analyses

HPLC-DAD analysis of anthocyanins was performed as described previously [18]. Briefly, separations were achieved at 45°C on a Luna C18(2), 4.6 × 150 mm, particle size 5 μm fitted with a corresponding guard-column (Phenonenex Inc, Santa Ana, CA) using an Agilent 1100 series (Agilent Technologies Inc., Montreal, QC, Canada) equipped with an autosampler with a 100 μL loop, a quaternary pump (maximum pressure, 400 bars), a column thermostat, and a diode array detector (DAD). The mobile phase consisted of 5% HCO_2_H in H_2_O (solvent A) and MeOH (solvent B). Optimized elution conditions were a linear gradient of 10-100% B in 25 min with a flow rate of 1 ml min^-1^, the column was washed for 10 min with 100% B, brought back to starting mobile phase composition in 0.1 min and equilibrated for 5 min prior to the next injection. The HPLC separations were monitored at 520, 476, 350, 280, and 254 nm. The relative quantities of anthocyanins were calculated based on percent area of each peak eluting at specific retention times.

The HPLC-ESI-MS/MS system consisted of an Agilent 1200 series (Agilent Technologies Inc., Montreal, QC, Canada) connected directly to a 3200QTRAP (ABI Sciex, Toronto, Canada). The software used for data acquisition and analysis is Analyst 1.4.2. The chromatographic conditions were the same as described for the HPLC-DAD analysis anthocyanins. The mobile phase was split 50/50 post-column. For enhanced mass scan (EMS), the MS was operated in positive polarity at a scan rate of 4000 amu s^-1 ^within the mass range of 100 - 1000 amu. The optimal source conditions of the mass spectrometer were: collision gas high, curtain gas (N2) 25 L min^-1^, ion spray voltage 4500 V, source temperature 500°C, source gas 1 (at 50 psig), source gas 2 (at 55 psig). The optimal compound parameters were declustering potential (DP) +20 V, entrance potential (EP) +10 V, collision energy (CE) 10 V, ionization energy 1.0 eV and detector 2800. For enhanced product ion scan the information dependant acquisition (IDA) criteria was as follows: select 1 - 2 most intense peaks which exceeds 10,000 cps, exclude targeted ions after 3 occurrences for 12 s. Product ions were scanned within the mass range of 100 - 1000 amu. All source parameters were the same as for EMS. The optimal compound parameters were DP +70 V, EP +10 V, CE 65 V, and collision energy spread 40 V.

## Authors' contributions

NK performed the molecular genetics experiments, the targeted analysis of anthocyanins and proanthocyanidins, assisted AM in performing the non-targeted metabolite analysis using LC-MS/MS, and wrote the manuscript. BM and JTA revised the manuscript and acquired the funding to support the research. All authors read and approved of the manuscript.

## Supplementary Material

Additional file 1 **Supplementary Table S1**. Developmental properties of black (iRT) and brown (irT) soybean seed coats at the 400 mg fresh seed weight stage. Click here for file

Additional file 2 **Supplementary Figure S1**. Proanthocyanidin (PA) subunit compositions, degree of polymerizations, and amounts from the seed coats of black (*iRT*) and brown (*irT*) soybean Clark isolines. (A, B, C) *iRT *top panels, *irT *bottom panels. (A) Phloroglucinol cleavage products of soluble PA polymers. HPLC retention times: ascorbic acid (1) (Rt: 2.0 min); phloroglucinol (2) (Rt: 3.1 min); epicatechin-phloroglucinol adduct (Rt: 5.5 min); epicatechin (4) (Rt: 9.4 min). (B) Phloroglucinol cleavage products of solvent insoluble PA polymers. HPLC retention times: ascorbic acid (1) (Rt: 2.0 min); phloroglucinol (2) (Rt: 3.1 min); epicatechin-phloroglucinol (Rt: 6.2 min); epicatechin (4) (Rt: 11.0 min). (C) Free monomers. HPLC retention times: epicatechin (1) (Rt: 12.5 min). (D) Mean degree of polymerization (mDP) of soluble PAs. (E) Total insoluble PAs. (E) Total soluble PAs. (D, E, F) *iRT *black bars, *irT *white bars. (E, D) Amounts are represented as milligrams procyanidin B2 equivalents per gram lyophilized seed coat (LSC). Click here for file

Additional file 3 **Supplementary Table S2**. Gene probe sets up-regulated more than 2-fold in the seed coat of black (iRT) soybean compared to the brown (irT) isoline^a^. Click here for file

Additional file 4 **Supplementary Figure S2**. Distribution of gene function categories of probe sets that were up-regulated more than 2-fold in the seed coat of black (*iRT*) soybean relative to the seed coat of brown (*irT*) soybean. Click here for file

Additional file 5 **Supplementary Table S3**. Glycine max UGTs^a,b^. Click here for file

Additional file 6 **Supplementary Table S4**. Glycine max OMTs^a,b^. Click here for file

Additional file 7 **Supplementary Table S5**. Gene probe sets downregulated more than 2-fold in the seed coats of black (iRT) soybean compared to the seed coats of brown (irT) soybean at the 300 - 400 mg fresh seed weight stage of development^a^. Click here for file

Additional file 8 **Supplementary Figure S3**. Distribution of gene function categories of probe sets that were down-regulated more than 2-fold in the seed coat of black (*iRT*) soybean relative to the seed coat of brown (*irT*) soybean. Click here for file

Additional file 9 **Supplementary Table S6**. Genes located on chromosome 9 of the soybean genome sequence Glyma1 that are associated with probe sets differentially-regulated more than 2-fold in the seed coat of black (iRT) soybean variety Clark^a^. Click here for file

Additional file 10 **Supplementary Figure S4**. Semi-qRT-PCR validation of the expressions of select genes found to be differentially expressed in black (*iRT*) and brown (*irT*) seed coats by microarray analysis. An asterix (*) represents genes located on Chromosome Gm09 of the soybean Glyma1 genome sequence. Genes and corresponding differentially regulated probe sets: 4CL-L, Gma.7423.2.S1_a_at; 4CL-2, Gma.8472.1.S1_at; CHS4 and CHS5, GmaAffx.42116.1.S1_at; LAR1, GmaAffx.34868.1.A1_at; DFR2, GmaAffx.80720.1.S1_at; ANS2/ANS3, Gma.1163.1.S1_at; UGT78K1, Gma.1002.2.S1_at; UGT78K2, GmaAffx.71999.1.S1_at; OMT-like, Gma.9647.1.S1_at; OMT5, GmaAffx.57777.1.S1_at; GST26, GmaAffx.71212.1.A1_at; GST21, Gma.5139.1.S1_at; MYB50, GmaAffx.81605.1.S1_at; MYB159, GmaAffx.39483.1.A1_at; C2H2 ZF, Gma.17736.1.S1_at; WD40, GmaAffx.45454.1.S1_at; SCOF-1, Gma.235.1.S1_at; AP2, GmaAffx.2469.1.S1_at; EF-hand, Gma.15972.1.A1_at; ProtK, GmaAffx.90491.1.A1_s_at; LRR, GmaAffx.12723.1.A1_at; G4DT, Gma.5621.1.S1_at; PAO1-L, Gma.3745.1.S1_at; Am Oxy, Gma.3745.1.S1_at; PCT, GmaAffx.78720.2.S1_at; PUB22, Gma.4530.1.A1_s_at; 9O12a, Gma.2605.1.S1_at; 9012b, Gma.2605.2.S1_at; Lipase, GmaAffx.90450.1.S1_at. Click here for file

Additional file 11 **Supplementary Figure S5**. Alignment of *G*. *max UF3GT* proteins UGT78K2 and UGT78K1 from variety Clark using the ClustalW program with default parameters. Amino acid differences are shown with grey background. Click here for file

Additional file 12 **Supplementary Table S7**. Primers. Click here for file
