# Supplementary figures and images for: Combined analysis of transcriptome and metabolite data reveals extensive differences between black and brown nearly-isogenic soybean (Glycine max) seed coats enabling the identification of pigment isogenes
Source: BMC Genomics. 2011 Jul 29;12:381. doi: 10.1186/1471-2164-12-381 (PMC3163566; doi:10.1186/1471-2164-12-381)

## Slide 1
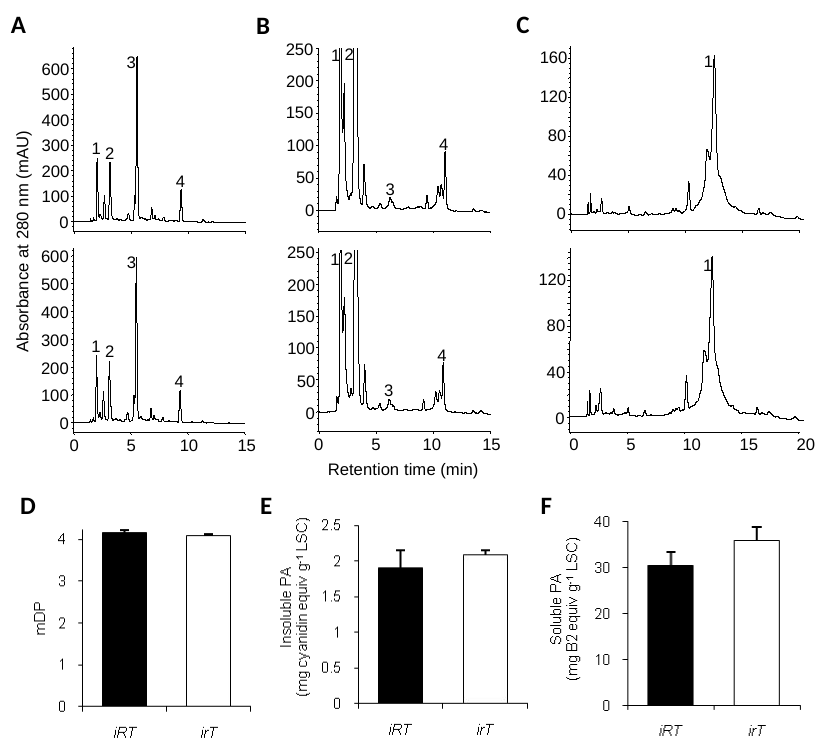

250
200
150
100
50
0
250
200
150
100
50
0
0
5
10
15
A
C
B
160
600
500
120
400
80
300
200
40
100
0
0
Absorbance at 280 nm (mAU)
600
120
500
400
80
300
200
40
100
0
0
0
5
10
15
20
0
5
10
15
2
1
1
3
4
1
2
4
3
2
1
3
1
1
2
4
4
3
Retention time (min)
D
F
E

Supplement: Additional file 2 — Supplementary Figure S1. Proanthocyanidin (PA) subunit compositions, degree of polymerizations, and amounts from the seed coats of black (iRT) and brown (irT) soybean Clark isolines. (A, B, C) iRT top panels, irT bottom panels. (A) Phloroglucinol cleavage products of soluble PA polymers. HPLC retention times: ascorbic acid (1) (Rt: 2.0 min); phloroglucinol (2) (Rt: 3.1 min); epicatechin-phloroglucinol adduct (Rt: 5.5 min); epicatechin (4) (Rt: 9.4 min). (B) Phloroglucinol cleavage products of solvent insoluble PA polymers. HPLC retention times: ascorbic acid (1) (Rt: 2.0 min); phloroglucinol (2) (Rt: 3.1 min); epicatechin-phloroglucinol (Rt: 6.2 min); epicatechin (4) (Rt: 11.0 min). (C) Free monomers. HPLC retention times: epicatechin (1) (Rt: 12.5 min). (D) Mean degree of polymerization (mDP) of soluble PAs. (E) Total insoluble PAs. (E) Total soluble PAs. (D, E, F) iRT black bars, irT white bars. (E, D) Amounts are represented as milligrams procyanidin B2 equivalents per gram lyophilized seed coat (LSC). [file 1471-2164-12-381-S2.PPT]

## Slide 1
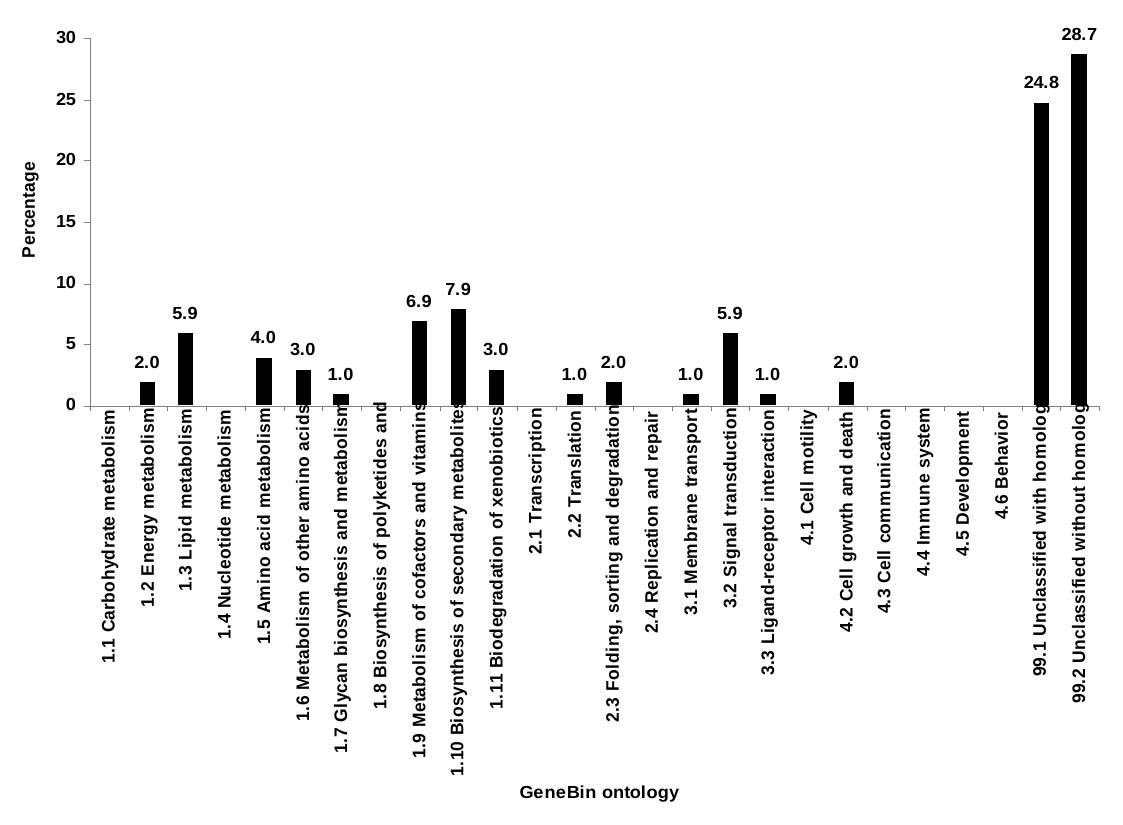

Supplement: Additional file 4 — Supplementary Figure S2. Distribution of gene function categories of probe sets that were up-regulated more than 2-fold in the seed coat of black (iRT) soybean relative to the seed coat of brown (irT) soybean. [file 1471-2164-12-381-S4.PPT]

## Slide 1
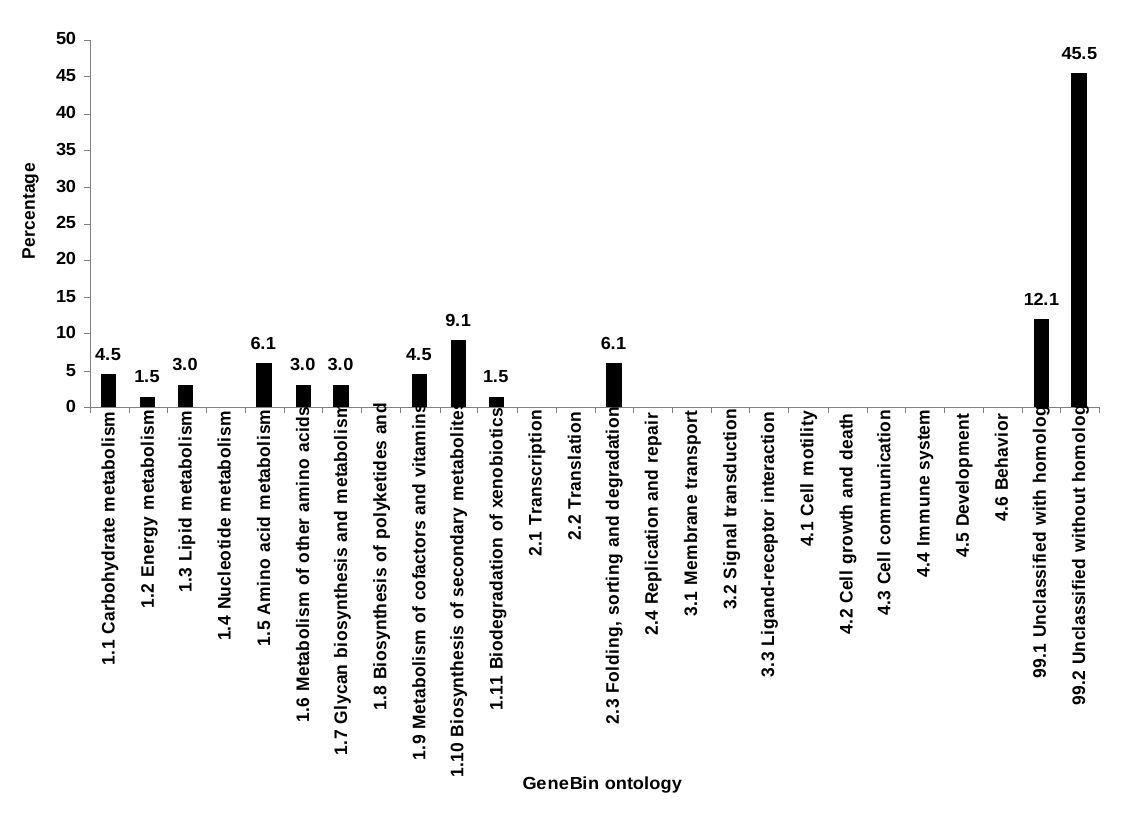

Supplement: Additional file 8 — Supplementary Figure S3. Distribution of gene function categories of probe sets that were down-regulated more than 2-fold in the seed coat of black (iRT) soybean relative to the seed coat of brown (irT) soybean. [file 1471-2164-12-381-S8.PPT]
