# Supplementary material for: Combined analysis of transcriptome and metabolite data reveals extensive differences between black and brown nearly-isogenic soybean (Glycine max) seed coats enabling the identification of pigment isogenes
Source: BMC Genomics. 2011 Jul 29;12:381. doi: 10.1186/1471-2164-12-381 (PMC3163566; doi:10.1186/1471-2164-12-381)
Supplement: Additional file 10 — Supplementary Figure S4. Semi-qRT-PCR validation of the expressions of select genes found to be differentially expressed in black (iRT) and brown (irT) seed coats by microarray analysis. An asterix (*) represents genes located on Chromosome Gm09 of the soybean Glyma1 genome sequence. Genes and corresponding differentially regulated probe sets: 4CL-L, Gma.7423.2.S1_a_at; 4CL-2, Gma.8472.1.S1_at; CHS4 and CHS5, GmaAffx.42116.1.S1_at; LAR1, GmaAffx.34868.1.A1_at; DFR2, GmaAffx.80720.1.S1_at; ANS2/ANS3, Gma.1163.1.S1_at; UGT78K1, Gma.1002.2.S1_at; UGT78K2, GmaAffx.71999.1.S1_at; OMT-like, Gma.9647.1.S1_at; OMT5, GmaAffx.57777.1.S1_at; GST26, GmaAffx.71212.1.A1_at; GST21, Gma.5139.1.S1_at; MYB50, GmaAffx.81605.1.S1_at; MYB159, GmaAffx.39483.1.A1_at; C2H2 ZF, Gma.17736.1.S1_at; WD40, GmaAffx.45454.1.S1_at; SCOF-1, Gma.235.1.S1_at; AP2, GmaAffx.2469.1.S1_at; EF-hand, Gma.15972.1.A1_at; ProtK, GmaAffx.90491.1.A1_s_at; LRR, GmaAffx.12723.1.A1_at; G4DT, Gma.5621.1.S1_at; PAO1-L, Gma.3745.1.S1_at; Am Oxy, Gma.3745.1.S1_at; PCT, GmaAffx.78720.2.S1_at; PUB22, Gma.4530.1.A1_s_at; 9O12a, Gma.2605.1.S1_at; 9012b, Gma.2605.2.S1_at; Lipase, GmaAffx.90450.1.S1_at. [file 1471-2164-12-381-S10.PPT]

## Slide 1
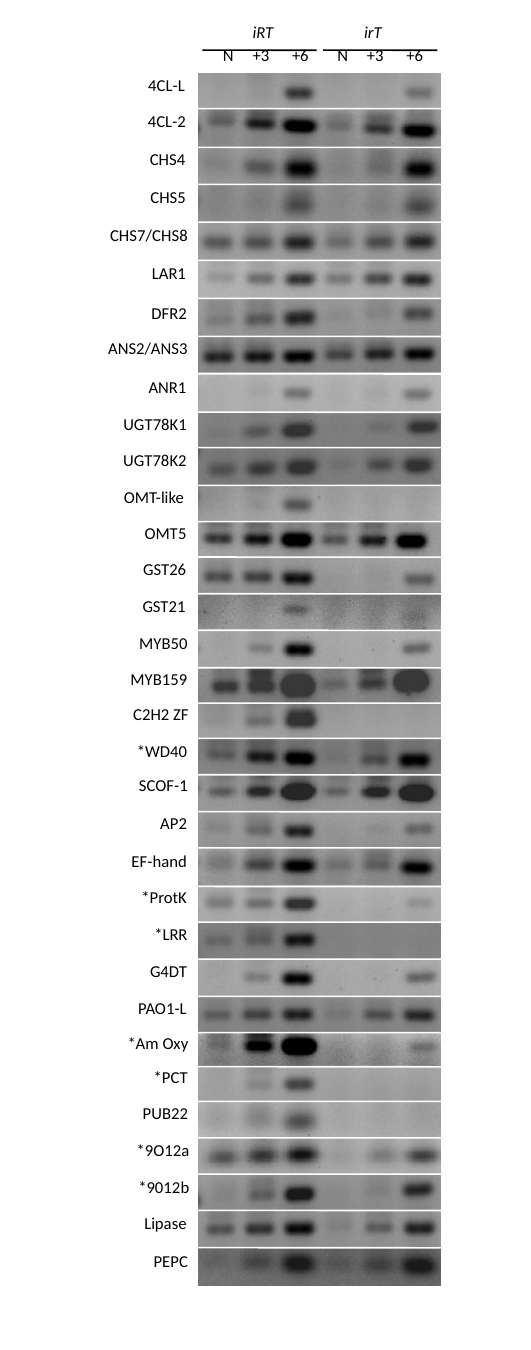

iRT
irT
N
+3
+6
N
+3
+6
4CL-L
4CL-2
CHS4
CHS5
CHS7/CHS8
LAR1
DFR2
ANS2/ANS3
ANR1
UGT78K1
UGT78K2
OMT-like
OMT5
GST26
GST21
MYB50
MYB159
C2H2 ZF
*WD40
SCOF-1
AP2
EF-hand
*ProtK
*LRR
G4DT
PAO1-L
*Am Oxy
*PCT
PUB22
*9O12a
*9012b
Lipase
PEPC
