# Supplementary material for: Combined analysis of transcriptome and metabolite data reveals extensive differences between black and brown nearly-isogenic soybean (Glycine max) seed coats enabling the identification of pigment isogenes
Source: BMC Genomics. 2011 Jul 29;12:381. doi: 10.1186/1471-2164-12-381 (PMC3163566; doi:10.1186/1471-2164-12-381)
Supplement: Additional file 11 — Supplementary Figure S5. Alignment of G. max UF3GT proteins UGT78K2 and UGT78K1 from variety Clark using the ClustalW program with default parameters. Amino acid differences are shown with grey background. [file 1471-2164-12-381-S11.DOC]

UGT78K2 MDHQNKHVAVFAFPFGSHLMPLLNLVLKLAHSLPNCSFSFIGTDKSNAILFPKPHIPNNI 60

UGT78K1 MDHQNKHVAVFAFPFGSHLMPLLNLVLKLAHSLPNCSFSFIGTHKSNAILFPKPHIPNNI 60

UGT78K2 KAYSISDGIPEGHVLGKNPTEKLNLFLQTGPENLHKGIELAEAETKKRVTCIVADAFVTS 120

UGT78K1 KAYSISDGIPEGHVLGKNPTEKLNLFLQTGPENLHKGIELAEAETKKRVTCIIADALVTS 120

UGT78K2 SLFVAQTLNVPWIALWLPNSCSLSLYFYTELIRQHCANHAGNTTLDFLPGLSKLRVEDMP 180

UGT78K1 SLLVAQTLNVPWIALWLPNSCSLSLYFYTDLIRQHCASRAGNKTLDFIPGLSKLRVEDMP 180

UGT78K2 QDLLDVGEKETVFARELNSLGKVLPQAKVVVMNFFEELEPPLFVQDMRSKLQSLLYVVPL 240

UGT78K1 QDLLDVGEKETVFSRELNSLGKVLPQAKVVVMNFFEELEPPLFVQDMRNKLQSLLYVVPL 240

UGT78K2 PSTLLPPSDTDSSGCLSWLDTKNSKSVAYVCFGTVVAPPPHELVAVAEALEESGFPFLWS 300

UGT78K1 PSTLLPPSDTDSSGCLSWLGMKNSKSVAYVCFGTVVAPPPHELVAVAEALEESGFPFLWS 300

UGT78K2 LKEGLIGLLPNGFVERTKKHGKIVSWAPQTQVLAHDSVGVFVTHCGANSVIESVSSGVPM 360

UGT78K1 LKEGLMSLLPNGFVERTKKRGKIVSWAPQTHVLAHDSVGVFVTHCGANSVIESVSSGVPM 360

UGT78K2 ICKPFFGDQVVAARVIEDVWEIGVIMEGKVFTKNGLVKSLDLILVHQEGKKIRDNALKVK 420

UGT78K1 ICRPFFGDQGVVARVIEDVWEIGMMIEGKMFTKNGLVKSLNLILVHEEGKKIRDNALRVK 420

UGT78K2 KTVEDAGRPEGQAAQDFDTLVEVISRS 447

UGT78K1 KTVEDAGRPEGQATQDFNTLVEVISRS 447

**Figure S11.** Alignment of *G*. *max* UG3GT proteins UGT78K2 and UGT78K1 from variety Clark using the ClustalW program with default parameters. Amino acid differences are shown with grey background.
